# Shen-Ling-Bai-Zhu-San Improves Dextran Sodium Sulfate-Induced Colitis by Inhibiting Caspase-1/Caspase-11-Mediated Pyroptosis

**DOI:** 10.3389/fphar.2020.00814

**Published:** 2020-05-29

**Authors:** Limin Chao, Zengquan Li, Jiahao Zhou, Wenqian Chen, Yuefei Li, Weijie Lv, Ao Guo, Qian Qu, Shining Guo

**Affiliations:** College of Veterinary Medicine, South China Agricultural University, Guangzhou, China

**Keywords:** Shen-ling-bai-zhu-san, ulcerative colitis, mitogen-activated protein kinase pathways, NF-κB pathways, pyroptosis

## Abstract

The traditional Chinese medicine Shen-ling-bai-zhu-san (SLBZS) is described in “Tai Ping Hui Min He Ji Ju Fang.” SLBZS has been shown to be effective against many gastrointestinal diseases. The present study aimed to investigate the effect of SLBZS on experimental colitis in mice and to define the potential mechanisms. Our data suggest that compared to the model group, SLBZS treatment increases mouse body weight and colon length, decreases the DAI score, and improves colonic injury. SLBZS reduces the production of cytokines (IL-1β, IL-18, and TNF-α) in colon tissue and mouse colonic mucosal epithelial (MCME) cells. Mechanistically, SLBZS inhibits inflammation by inhibiting the MAPK and NF-κB signaling pathways. Further mechanistic analyses showed that SLBZS attenuates the expression levels of pyroptosis-related genes, including NLRP3, ASC, and GSDMD-N in the colons of mice. In addition, SLBZS restores the levels of the colon tight junction proteins ZO-1 and occludin, suggesting that it protects colonic barrier integrity and ameliorates the progression of colitis. In this paper, we demonstrate that SLBZS attenuates DSS-induced ulcerative colitis injury in mice *via* the MAPK/NF-κB and pyroptosis signaling pathway. These results indicate that SLBZS is a potential drug for the treatment of UC.

## Introduction

Ulcerative colitis (UC) is a nonspecific inflammatory disease that results from chronic inflammation and ulceration of the inner wall of the rectum and colon (Ungaro et al., 2017). UC usually starts at the rectum, involves the colonic mucosa and submucosa, and then spreads throughout the colon over time (Ordas et al., 2012). UC is characterized by bloody diarrhea, intestinal mucosal ulceration, and neutrophil and lymphocyte infiltration into the mucosa. Long-term UC patients have an increased risk of developing colorectal cancer (Polytarchou et al., 2015). A number of studies have linked UC with an increased risk of colon cancer and demonstrated that 40% of UC cases over 25 years eventually develop colorectal cancer (Shivashankar et al., 2017). In the West, the incidence and prevalence of UC have increased in the past 50 years, affecting up to 6 to 15/100,000 and 50 to 200/100,000 persons. However, in recent years, with the popularization of the Western lifestyle, the incidence of UC has begun to the rise in developing nations, such as India and China (Cosnes et al., 2011). With its increased incidence and characteristics, the World Health Organization (WHO) lists UC as an incurable disease (Zhang et al., 2014).

Pyroptosis is a form of cell death in which cells swell, and large blebs appear in the plasma membrane when pyroptosis occurs (Duprez et al., 2009). Studies have reported that the downregulation of the pyroptosis signaling pathway can improve experimental colitis (Xiong et al., 2016). Studies have shown that the mitogen-activated protein kinase (MAPK) pathway is a prominent signal transduction pathway for colitis injury and plays important roles in regulating cell proliferation, differentiation, and apoptosis (Cao et al., 2018). Nuclear factor-kappa B (NF-κB) and inflammatory bodies are key components of inflammation, and their dysregulation promotes the production of UC-associated proinflammatory cytokines (Cui et al., 2019). NF-κB is a transcription factor involved in the production and release of proinflammatory cytokines and chemokines (Palmela et al., 2015).

SLBZS is a typical Chinese medicine spleen prescription for treating diarrhea and loss of appetite and was first proposed in “Tai Ping Hui Min He Ji Ju Fang” in the Song Dynasty (Yang et al., 2018). It has been reported that SLBZS can alleviate lactose-induced chronic diarrhea in rats (Ji et al., 2019). A study found that SLBZS can improve the immunosuppressive tumor microenvironment and reduce the incidence of colon tumors (Lin et al., 2015). However, there is a lack of research regarding its role in the recovery of UC induced by DSS.

Although several drug interventions have been studied to alleviate the symptoms of UC patients, most of these treatments have serious side effects (Bressler et al., 2015). Scientists are beginning to focus on natural drugs with fewer side effects than current treatments (Montrose et al., 2011). Therefore, the purpose of this study was to investigate the mechanism by which SLBZS alleviates DSS-induced UC in mice and further investigate whether it is related to the MAPK and NF-κB signaling pathways and pyroptosis.

## Materials and Methods

### Drugs

SLBZS consists of ten kinds of Chinese medicinal herbs, including *Panax ginseng C. A. Mey*. (4 g, Batch No: 20190609), *Poria cocos (Schw.) Wolf* (4 g, Batch No: 20191020), *Atractylodes macrocephala Koidz*. (4 g, Batch No: 19111603), *Dioscorea opposita Thunb*. (4 g, Batch No: 19051401), *Dolichos lablab L*. (3 g, Batch No: 19091703), *Nelumbo nucifera Gaertn*. (2 g, Batch No: 19040702), *Coix lacryma-jobi L*. var. *mayuen. (Roman.) Stapf* (2 g, Batch No: 19092305), *Amomum villosum Lour*. (2 g, Batch No: 20191017), *Platycodon grandiflorum (Jacq.) A. DC*. (2 *g*, Batch No: 19042001), and *Glycyrrhiza glabra L*. (4 g, Batch No: 190723). All Chinese medicines were purchased from Tongren tang drug store (Guangzhou, Guangdong, China). All herbal materials were identified by Professor Shining Guo of South China Agricultural University. All of the samples were stored in the herbarium of South China Agricultural University (Herbarium Code: SCAUB), oucher numbers: *Panax ginseng C. A. Mey*. (198926), *Poria cocos (Schw.) Wolf* (00559936), *Atractylodes macrocephala Koidz*. (1899007), *Dioscorea opposita Thunb*. (663941), *Dolichos lablab L*. (2501864), *Nelumbo nucifera Gaertn*. (2608713), *Coix lacryma-jobi L*. var. *mayuen. (Roman.) Stapf* (652488), *Amomum villosum Lour*. (3777516), *Platycodon grandiflorum (Jacq.) A. DC*. (3501015), *Glycyrrhiza glabra L*. (703906). A Chinese medicine pulverizer was used for powdering, and the SLBZS was then filtered through a sieve (40–60 mesh). The SLBZS was diluted 10 times and cooked for 30 min; this step was repeated twice. The supernatant was collected and concentrated and then filtered using a 0.22-μm filter. The supernatant of SLBZS was stored in a −20°C refrigerator, and it was cooked every seven days.

### Experimental Animals

Fifty males C57BL/6 mice weighing 20 to 24 g were purchased from Speyer (Beijing) Biotechnology Co., Ltd. According to the guidelines of the Experimental Animal Center of South China Agricultural University, all animals were kept in-house, housed in specific pathogen-free animal facilities, with a 12-h light/dark cycle per day and constant temperature (25 ± 3 °C) and humidity (65 ± 10%), and fed standard laboratory chow. The program was approved by the Animal Experimental Ethics Review Committee of South China Agricultural University.

### Experimental Design and Drug Treatment

The study was divided into two stages: a DSS treatment stage and a drug treatment stage. First, mice (n = 50) were assigned to control (control, n = 10) and DSS (n = 40) groups. The colitis was induced in the DSS group by the administration of 3% DSS (molecular weight: 40,000 Da; KINGBIO, USA), in the drinking water for 7 days. Then, the DSS group mice (n = 40) were equally assigned into model (model), low-dose (L-dose), medium-dose (M-dose) and high-dose (H-dose) groups. The control and model groups were given daily drinking water, and the treatment groups were orally administered with SLBZS 1.183 g/kg, 2.366 g/kg, and 4.732 g/kg. The specific experimental steps are shown in **Figure 2A**. At the end of the experiment, after fasting for 24 h, blood was collected *via* the orbital sinus, and centrifuged at 4°C for 10 min; the supernatant was stored at −80°C. Then, the mice were euthanized, and the liver, spleen, kidney, and colon were collected. Colon lengths were recorded, and colon specimens were frozen in liquid nitrogen or immediately fixed in 10% (w/v) formalin solution for further analysis.

### Analysis of the Main Components of SLBZS

The five chemical components of SLBZS were analyzed by a Waters 1525 high-performance liquid chromatography (HPLC) system. SLBZS components were separated by a Sunfire C18 (4.6 mm × 250 mm, 5 μm). For each run, 10 μl was taken, and the flow rate was 1.0 ml/min. Ginsenoside Rg1(Solarbio, 724B021), ginsenoside Re (Solarbio, 717B021), ginsenoside Rb1(Solarbio, 727C021), liquiritin (Meilunbio, A0802AS), and atractylenolide III (Meilunbio, O1021AS) were selected as the standard products and were detected at a wavelength of 203 nm. The mobile phase was 0.05% phosphoric acid (A) and acetonitrile (B). The gradient procedure was as follows: 79% for 0 to 30 min; 79% to 71% for 30 to 50 min; 71% for 50 to 70 min; 71% to 52% for 70 to 100 min; and 52% to 52% for 100 to 130 min.

### Observation of UC Symptoms and Signs in Mice

At the end of the experiment, the alleviation of UC in mice by SLBZS treatment was evaluated. Indicators of colitis damage, including body weight, disease activity index (DAI), colon length, organ coefficient, hematology, and tissue section observation, were evaluated. DAI scores include weight loss scores, fecal status scores, and bloody stool scores. In short, the parameters were calculated according to the following criteria: weight loss score (0, no weight loss; 1, 1%−5%; 2, 6%−10%; 3, over 11%), stool status score (0: normal, 1: loose stool, 2: muddy feces, 3: diarrhea) and bloody stool scores (0: none, 1: occult blood, 2: bleeding, 3: gross bleeding).

### Analysis of Antioxidative Enzymatic Activity and Myeloperoxidase Activity

Briefly, approximately 150 mg of colon tissue from each group was weighed and homogenized with PBS [1:9 (w/v)]. According to the manufacturer’s instructions (Nanjing Institute of Bioengineering, China), the malondialdehyde (MDA) and superoxide dismutase (SOD) levels in the colon were analyzed. Myeloperoxidase (MPO) reflects the function and activity status of neutrophils in tissues (Shen et al., 2019). MPO levels were measured according to the manufacturer’s instructions for the MPO ELISA kit (Shanghai Enzyme-linked Biotechnology Co., Ltd., China).

### Histological Examination

The mouse colon was fixed in 10% formalin, embedded in paraffin and cut into sections. Then, the sections were stained with hematoxylin and eosin (H&E) and observed under light microscopy.

### Measurement of Cytokines

The colon was cut into small pieces and homogenized with iced-cold Tris-HCl buffer to extract total protein. The levels of IL-1β, IL-18, and TNF-α were determined by enzyme linked immunosorbent assay. All procedures followed the manufacturer’s guidelines.

### Caspase-1 and Caspase-11 Activity Assays

The key execution mechanism of pyroptosis involves the activation of inflammatory caspase-1 and nonclassical caspase-11 (Lamkanfi and Dixit, 2014; Yuan et al., 2016). The activity levels of caspase-1 and caspase-11 were measured using an activity assay kit according to the manufacturer’s instructions (Shanghai Enzyme-linked Biotechnology Co., Ltd., China). The caspase activity detection kit works by monitoring the caspase-induced production of yellow free nitroaniline pNA (p-nitroaniline) from acetyl-tyr-val-ala-asp pNA (ac-yvad-pNA). The caspase-1 and caspase-11 activity levels were indirectly detected by the colorimetric detection of absorbance.

### Western Blot Analysis

Colon tissue was homogenized in RIPA buffer (Beyotime, China)with phosphatase inhibitor (Beyotime, China). The colonic protein concentration was measured using a BCA Protein Assay kit (Beyotime, China). Protein lysates were separated by SDS-PAGE (Beyotime, China). The cells were transferred to a 0.22-μm PVDF membrane (Millipore Corporation, Billerica, USA). After blocking with 5% nonfat milk powder in PBS for 1.5 h, the membranes were incubated with p38(dilution 1:1000, Cell Signaling Technology, USA), p-p38 (dilution 1:1000, Cell Signaling Technology, USA), ERK1/2 (dilution 1:2000, Zen-bioscience, China), p-ERK1/2 (dilution 1:2000, Zen-bioscience, China), p65 (dilution 1:1000, Cell Signaling Technology, USA), p65 (dilution 1:1000, Cell Signaling Technology, USA), JNK (dilution 1:1000, Cell Signaling Technology, USA), p-JNK (dilution 1:1000, Cell Signaling Technology, USA), IKK-α/β (dilution 1:1000, Beyotime, China), p-IKK-α/β (dilution 1:1000, Affinity Biosciences, USA), NLRP3 (dilution 1:1000, Cell Signaling Technology, USA), GSDMD-N (dilution 1:1000, Santa Cruz Biotechnology, USA), ASC (dilution 1:1000, Cell Signaling Technology, USA), caspase-1 (dilution 1:1000, Cell Signaling Technology, USA), caspase-11 (dilution 1:1000, Santa Cruz Biotechnology, USA), ZO-1 (dilution 1:1000, ThermoFisher, USA), and occludin (dilution 1:1000, ThermoFisher, USA) primary antibodies overnight in a refrigerator at 4°C with shaking. After incubation with the primary antibodies, the membranes were washed 3 times (10 min/wash) with TBST. The secondary antibody (1:5000) was added, and the membranes were incubated for 1 h with shaking and then washed three times (10 min/wish) with TBST. The protein bands were visualized with ECL reagents (PerkinElmer, USA). The protein content was assessed by Image-Pro Plus software.

### Cell Culture and Viability Assay

Mouse colonic mucosal epithelial (MCME) cells (iCell Bioscience Inc, Shanghai, China) were cultured in iCell Primary Epithelial Cell Medium (iCell Bioscience Inc., Shanghai, China) supplemented with 10% FBS, penicillin (100 U/ml), and streptomycin (100 μg/ml). MCME cells in 25 cm^−2^ cell culture flasks were cultured in a humidified incubator at 37°C with 5% CO_2_.

MCME cell viability was measured by MTT assay. Cells (1×10^5^ cells/well) in a 90-μl volume were added to 96-well plates and cultured for 24 h. Then, 10 μl of MTT solution (5 mg/ml) was added, and the plate was incubated for 4 h at 37°C in a 5% CO _2_ incubator. After 100 μl of DMSO was added to each well, the absorbance at a wavelength of 570 nm was measured with a microplate (Multiskan MK3; Thermo Scientific Co., Ltd., USA) to calculate the cell growth inhibition rate.

### DSS+LPS-Induced Cell Inflammation Model

The cells were cultured in 100 μl of medium for 24 h with or without SLBZS (concentrations of 0.313%, 0.625% and 1.25%). Then, 100 μl of 10 μg/ml DSS was added to each well for 2 h. Subsequently, the supernatant was aspirated, the cells were washed with PBS, and 100 μl of 1 μg/ml LPS (Sigma Aldrich, St Louis, USA) was added and incubated with the cells for 12 h.

### Statistical Analysis

All data were performed using SPSS 20.0 (IBM, Armonk, NY, USA) software. GraphPad Prism 5.0 (GraphPad; San Diego, CA, USA) was used for statistical analysis. Results are shown as the mean ± S.E.M. The mean between groups was analyzed by one-way ANOVA and then Fisher’s LSD test. P < 0.05 was considered significant.

## Results

### SLBZS Chemical Component Analysis

**Figure 1:**
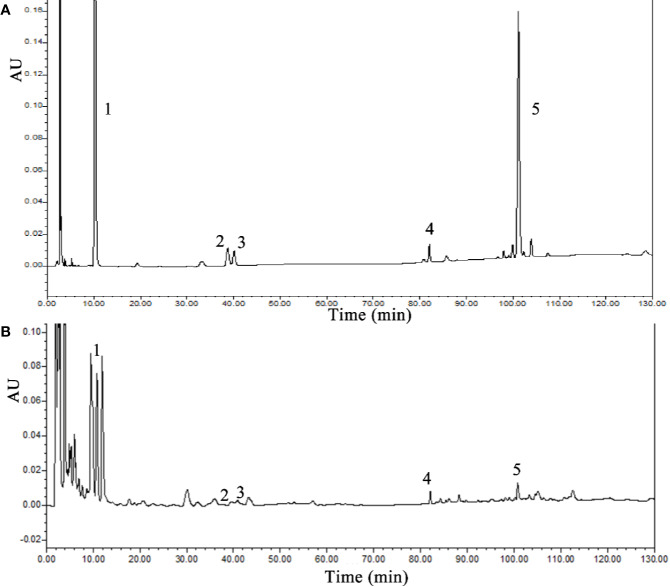
HPLC analysis of SLBZS. **(A)** SLBZS decoction. **(B)** Standards: 1, liquiritin; 2, ginsenoside Re; 3, ginsenoside Rg1; 4, ginsenoside Rb1; 5, atractylenolide III.

Five chemical components were identified by HPLC as components of SLBZS: liquiritin, ginsenoside Re, ginsenoside Rg1, ginsenoside Rb1, and atractylenolide III. The concentrations were 0.59, 0.21, 0.7, 0.92, and 0.15 mg/g, respectively (**Figures 1A, B**).

### SLBZS Significantly Alleviates DSS-Induced Colitis

**Figure 2:**
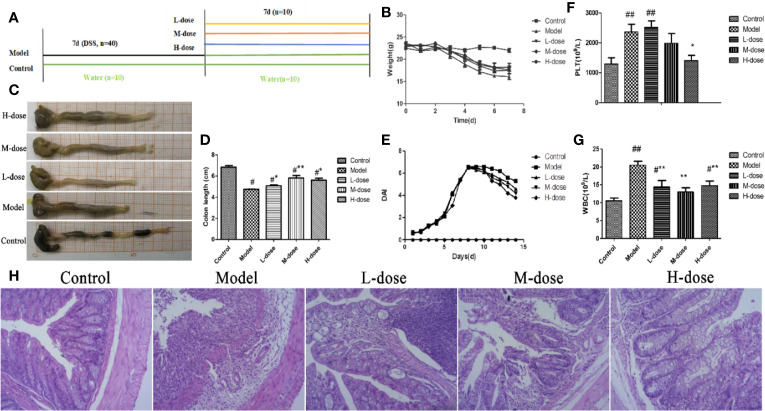
SLBZS ameliorates DSS-induced colonic inflammatory symptoms in mice. **(A)** The experimental scheme of SLBZS treatment in DSS-induced colitis in mice. **(B)** Body weight (n = 10). **(C)** Representative picture of the colon. **(D)** Colon length (n = 10). **(E)** DAI reflecting weight loss scores, fecal status scores, and bloody stool scores, assessed every day. **(F, G)** Routine blood tests for the number of PLTs and WBCs in the serum of mice. **(H)** Representative image of mouse colon stained with H&E (×100). The data are shown as the mean ± S.E.M. ^#^*P* < 0.05, ^##^*P* < 0.01 vs. the control group; ^*^P < 0.05, ^**^P < 0.01 vs. the model group.

To assess the therapeutic effect of SLBZS on 3% DSS-induced colonic epithelial damage and colitis in mice, we further determined whether SLBZS treatment improves colitis. Specific detailed steps were carried out according to the experimental design schematic (**Figure 2A**). The mouse 3% DSS-induced colitis model was used to investigate the effect of SLBZS on colitis. Compared with that of the control mice, the weight of the mice that received DSS was significantly decreased; after SLBZS administration, the body weights of the mice increased (**Figure 2B**). As shown in **Figures 2C, D**, DSS causes a shortening of the colon length. However, the administration of 1.183, 2.366, and 4.732 g/kg SLBZS significantly prevented the shortening of the colon. In addition, the DAI score and histological damage were decreased in colitis mice treated with SLBZS (**Figures 2E, H**). Hematological analyses revealed that compared with the control group, the DSS-induced colitis group had significant increases in absolute platelet (PLT) and white blood cell (WBC) counts (**Figures 2F, G**). Compared to the model group, the SLBZS treatment group had significantly reduced absolute numbers of PLTs and WBCs (**Figures 2F, G**).

### Effects of SLBZS on Oxidative Stress and MPO in Mouse Colon

**Figure 3:**
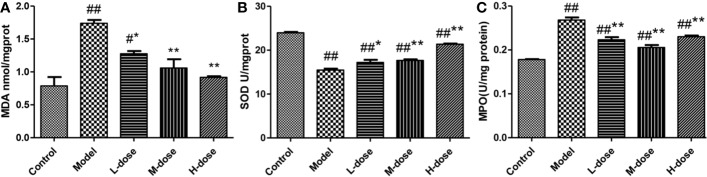
Effects of SLBZS on oxidative stress and MPO in the mouse colon. **(A, B)** MDA and SOD concentrations in the colon tissue. **(C)** MPO activity in the colon tissue. The data are shown as the mean ± S.E.M. ^#^*P* < 0.05, ^##^*P* < 0.01 vs. the control group; ^*^P < 0.05, ^**^P < 0.01 vs. the model group.

To assess the effects of SLBZS on colonic epithelial damage and neutrophil infiltration, MDA, SOD, and MPO levels were examined. DSS-induced oxidative stress levels in mice were examined according to the kit instructions. As shown in **Figures 3A, B**, compared with that in the model group, the level of MDA in the SLBZS treatment group was significantly decreased, and the levels of SOD were significantly increased, and oxidative stress levels were significantly reduced in the colon. SLBZS treatment significantly reduced MPO activity in DSS-induced colitis mice and increased MPO activity in the model group (**Figure 3C**). The results of this study indicate that SLBZS treatment protects against colitis in mice.

### SLBZS Regulates the MAPK and NF-κB Pathways

**Figure 4:**
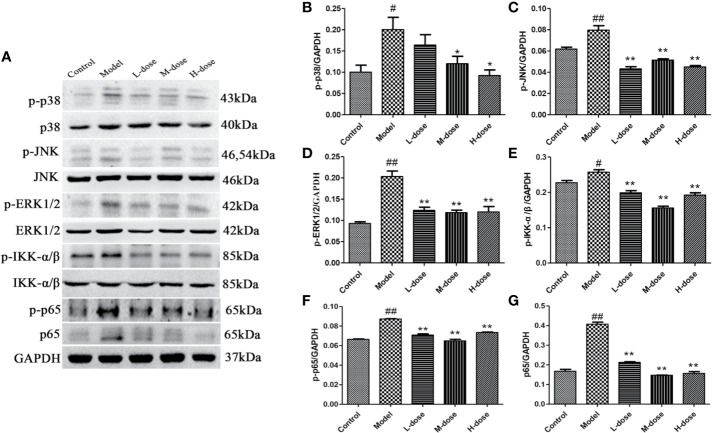
SLBZS regulates the MAPK and NF-κB pathways. **(A)** The expression levels of p-p38, p-JNK, p-ERK1/2, p-IKK-α/β, p-p65, and p65 were analyzed by western blotting. **(B**–**G)** Analysis of protein concentration. The data are shown as the mean ± S.E.M. ^#^*P* < 0.05, ^##^*P* < 0.01 vs. the control group; ^*^P < 0.05, ^**^P < 0.01 vs. the model group.

To investigate the mechanism of SLBZS in DSS-induced mouse colitis, the expression levels of the major proteins p-p38, p-ERK1/2, p-JNK, p-IKK-α/β, p-p65, and p65 in the MAPK and NF-κB signaling pathways were measured (**Figure 4A**). The experimental results showed that the phosphorylation levels of p38, JNK, ERK1/2, IKK-α/β were attenuated after stimulation with SLBZS, and the levels of p-p65 and p65 were simultaneously reduced (**Figures 4B–G**). These results indicate that SLBZS suppresses DSS-induced colitis inflammation by gulating activation of the MAPK and NF-κB signaling pathways.

### Effects of SLBZS on the Viability of MCME Cells

**Figure 5:**
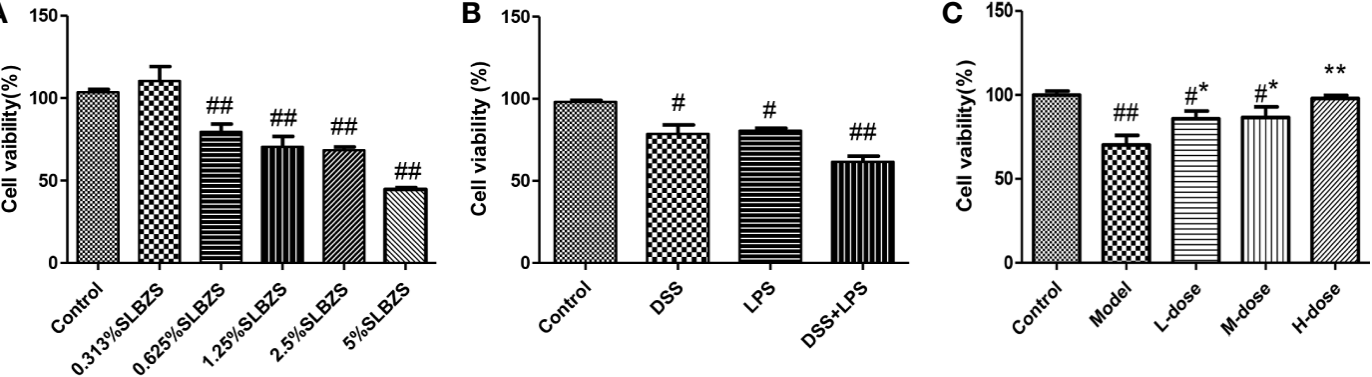
Effects of SLBZS on the viability of MCME cells. **(A)** MCME cells were cultured for 24 h in 0.313%, 0.625%, 1.25%, 2.5% and 5% SLBZS medium, and the viability of each group was measured by the MTT assay. **(B)** The MCME cells were cultured for 24 h in medium containing 10 μg/ml DSS or 1 μg/ml LPS, and the viability of each group was measured by the MTT assay. **(C)** The cells were cultured in 10 μg/ml DSS for 2 h, and then the supernatant was discarded, the cells were washed with PBS, and 1 μg/ml LPS was added to the medium for 12 h. Subsequently, the viability of each group was measured by the MTT assay. The data are shown as the mean ± S.E.M. ^#^*P* < 0.05, ^##^*P* < 0.01 vs. the control group; ^*^P < 0.05, ^**^P < 0.01 vs. the model group.

We primarily used the MTT assay to measure the survival rate of cells. As shown in **Figure 5A**, at an SLBZS concentration of 5%, the viability of more than 50% of the cells was inhibited. Therefore, concentrations of 0.313%, 0.625% and 1.25% SLBZS were used in the L-dose, M-dose, and H-dose groups, respectively. DSS combined with LPS had better effects on cell damage than DSS alone, so DSS in combination with LPS was used to induce cellular inflammation (**Figure 5B**). SLBZS exerts a significant protective effect against cell damage, especially in the H-dose group (**Figure 5C**).

### SLBZS Inhibits Proinflammatory Cytokine Production in Tissues and Cells

**Figure 6:**
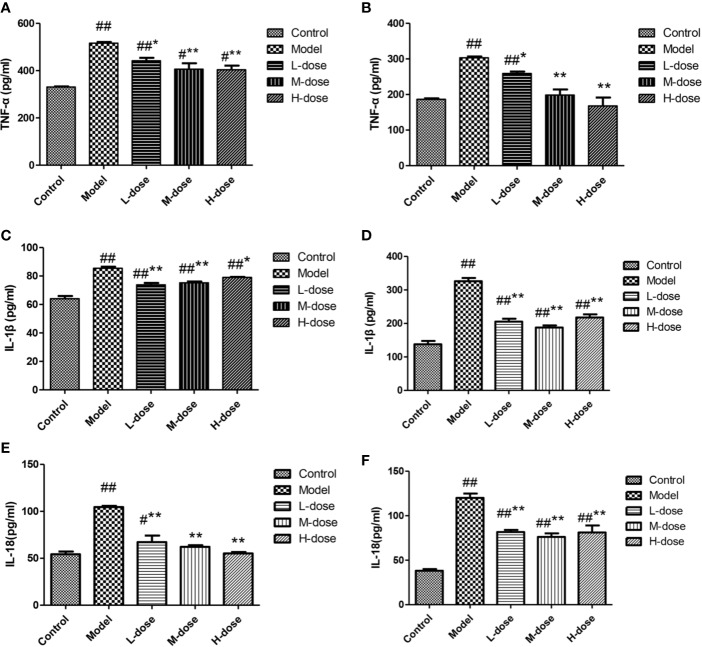
MCME cell was incubated with different concentrations of SLBZS for 24 h. Then, 100 μl of 10 μg/ml DSS was added to each well for 2 h. Subsequently, the supernatant was pipetted, and 100 μl of 1 μg/ml LPS was added and incubated with the cells for 12 h. The effect of SLBZS on proinflammatory factors. Commercial TNF-α, IL-1β, and IL-18 kits were used to measure colon tissue **(A, C, E)** and cellular **(B, D, F)** proinflammatory factor levels. The data are shown as the mean ± S.E.M. ^#^*P* < 0.05, ^##^*P* < 0.01 vs. the control group; ^*^P < 0.05, ^**^P < 0.01 vs. the model group.

The levels of cytokines were measured by an ELISA kit. Compared with the control group, the DSS-treatment group had significantly increased levels of the inflammatory cytokines TNF-α, IL-1β, and IL-18 (**Figures 6A–F**). SLBZS treatment significantly reduced the levels of the cytokines TNF-α, IL-1β, and IL-18 (**Figures 6A–F**). These experimental results show that SLBZS may play a direct role in the treatment of inflammatory processes.

### SLBZS Inhibits Pyroptosis

**Figure 7:**
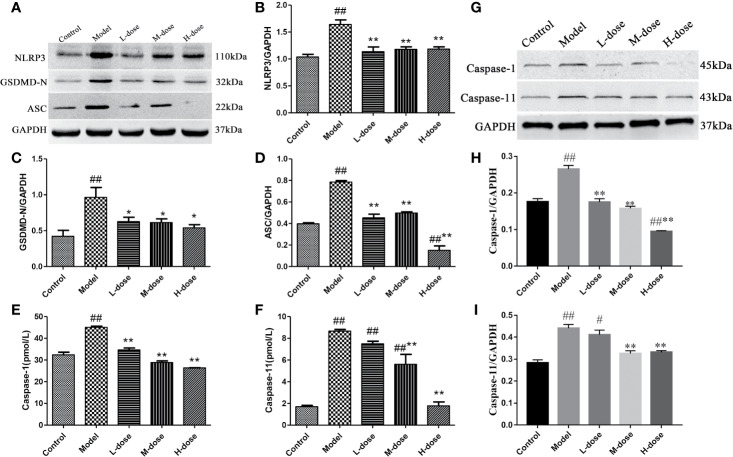
SLBZS reduces the progression of colitis by inhibiting pyroptosis. **(A–D)** The expression levels of NLRP3, GSDMD-N, and ASC were analyzed by western blotting. **(E**, **F)** A kit was used to measure caspase-1 and caspase-11 levels. **(G**–**I)** Protein expression of caspase-1 and caspase-11. The data are shown as the mean ± S.E.M. ^#^*P* < 0.05, ^##^*P* < 0.01 vs. the control group; ^*^P < 0.05, ^**^P < 0.01 vs. the model group.

Pyroptosis is a type of programmed cell death (PCD) that mediates cell death primarily during inflammatory processes (Yu et al., 2019). Therefore, to investigate whether SLBZS exerts anti-inflammatory effects by inhibiting pyroptosis, we measured the protein levels of NLRP3, GSDMD-N, and ASC by Western blot and caspase-1 and caspase-11 levels were detected by commercial kits. The results indicated that compared with the model group the expression levels of NLRP3, GSDMD-N, and ASC protein were significantly decreased in the SLBZS-treatment groups, especially in the H-dose group (**Figures 7A–D**). As shown in **Figures 7E, F**, the caspase-1 and caspase-11 levels were significantly decreased. Furthermore, Western blot detection revealed that SLBZS can reduce caspase-1 and caspase-11 enzyme activities (**Figures 7G–I**). These experimental results show that SLBZS reduces the progression of colitis by inhibiting pyroptosis.

### SLBZS Modulates the Expression of ZO-1 and Occludin in the Colonic Mucosa

**Figure 8:**
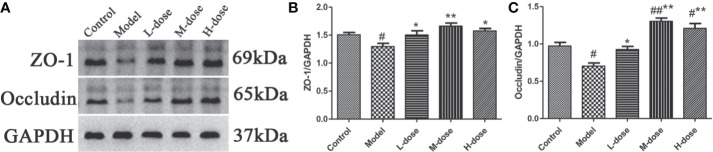
SLBZS protects the colonic mucosal barrier integrity of mice. **(A**–**C)** ZO‐1 and occludin expression levels in the colon were analyzed by western blotting. The data are shown as the mean ± S.E.M. ^#^*P* < 0.05, ^##^*P* < 0.01 vs. the control group; ^*^P < 0.05, ^**^P < 0.01 vs. the model group.

Studies have shown that intestinal inflammation is associated with intestinal mucosal permeability caused by intestinal barrier damage (Zhang et al., 2018). ZO-1 and occludin are important colonic mucosal proteins that maintain the integrity of the colonic mucosal barrier (Fries et al., 2013; Ji et al., 2016). To further verify the protective effect of SLBZS on the colon, the expression levels of ZO-1 and occludin were examined. We found that the levels of ZO-1 and occludin were significantly decreased in the model group compared with the control group (**Figures 8A–C**). However, the expression levels of ZO-1 and occludin proteins were increased after SLBZS treatment (**Figures 8A–C**). These results indicate that SLBZS protects the colonic mucosal barrier integrity and can alleviate colitis.

## Discussion

UC is an autoimmune disease caused by excessive activation of the immune system. DSS-induced colitis is typically used in animal model of UC (Bramhall et al., 2015). DSS can disrupt the integrity of the intestinal epithelial barrier and increase epithelial permeability, leading to intestinal mucosal antigens and microbes entering the mucosa, and causing the infiltration of immune cells, such as macrophages and neutrophils into the lamina propria and submucosa; in addition, DSS causes intense inflammation and the release of many proinflammatory cytokines (Gao et al., 2018). In this study, we demonstrate for the first time the alleviation of 3% DSS-induced acute colitis by SLBZS treatment in mice. Treatment with SLBZS reduced the mouse weight loss and colon length shortening and improved DAI scores, suggesting that SLBZS has therapeutic potential for colitis. Our study shows that DSS-induced MAPK and NF-κB signaling is inhibited by SLBZS treatment. In addition, we found that SLBZS plays a key role in relieving inflammation and maintaining intestinal integrity by inhibiting pyroptosis and maintaining intestinal permeability.

*Panax ginseng C. A. Mey*., *Atractylodes macrocephala Koidz*., and *Glycyrrhiza glabra L*. are among the main SLBZS drugs. It has been reported that ginsenoside Rg1 in Panax ginseng C. A. Mey. gulates NLRP12, inhibits the release of IL-1β and TNF-α, and reduces DSS-induced inflammatory response in colitis in mice (Zhu et al., 2017). Atractylodes III in Atractylodes macrocephala Koidz. macrocephala inhibits the production of TNF-α and NO in macrophages induced by LPS (Li et al., 2007). Glycyrrhizin in Glycyrrhiza glabra L. can reduce the inflammatory response, oxidative stress, and apoptosis by inhibiting the activation of TLR4/MyD88/NF-κB and gulating the release of oxidants (Li et al., 2018).

Our experimental results demonstrate that SLBZS has a therapeutic effect on DSS-induced colitis in mice. The experimental colitis model is characterized by weight loss, bloody diarrhea, inflammatory mediator production, and intestinal mucosal immune cell infiltration (Gao et al., 2019; Zhang et al., 2019). However, there is currently no high-quality study of SLBZS in DSS-induced colitis. In this study, compared to control mice, DSS-treated mice showed sustained weight loss, colon shortening, inflammatory cell infiltration, and increased histological scores. However, SLBZS treatment improved this situation. Furthermore, the reduction in MPO activity by SLBZS improved colitis. Hematological examinations revealed significant changes in PLT and WBC counts. Moreover, SLBZS treatment significantly improved the levels of MDA and SOD in the colon and inhibited oxidative stress in the colon. These experimental results show that as the SLBZS concentration increases, the treatment effect improves accordingly, indicating that SLBZS may be a potential drug for the treatment of colitis.

We studied the underlying mechanisms of SLBZS-mediated inflammation alleviation. MAPK plays key roles in the regulation, proliferation, and differentiation of proinflammatory cells (Kyriakis and Avruch, 2012). NF-κB regulates gene expression in proinflammatory mediators and plays an important role in the initiation of experimental colitis (Zou et al., 2016). Clinical studies report excessive or abnormal activation of NF-κB in patients with IBD (Hu et al., 2018). Our study demonstrates that SLBZS can inhibit the expression levels of p-p38 and p-ERK1/2 and NF-κBp65 proteins in colitis mice. Furthermore, our results indicate that SLBZS alleviates colitis in mice by gulating the activation of the MAPK and NF-κB signaling pathways.

We demonstrate *in vitro* that SLBZS has a protective effect on LPS-induced MCME cell inflammation. The levels of the inflammatory factors TNF-α, IL-1β, and IL-18 were detected in tissues and cells, and the results showed that SLBZS treatment significantly inhibited the release of inflammatory mediators and reduced UC symptoms. Pyroptosis is an inflammation-mediated type of PCD, and the release of IL-1β and IL-18 can amplify the inflammatory response (Duprez et al., 2009; Shi et al., 2015). Pyroptosis is mainly caused by the caspase-1 activation pathway in typical inflammation and the caspase-11 pathway in nonclassical inflammation (Demon et al., 2014; Liu et al., 2016). We found that SLBZS treatment reduces the protein levels of NLRP3, GSDMD-N, ACS, caspase-11, and IL-1β. Thus, SLBZS relieves colonic inflammation by reducing caspase-1/-11 levels and inhibiting pyroptosis.

The intestinal mucosal barrier maintains homeostasis and prevents the invasion of antigens from the external environment (Turner and , 2009; Martini et al., 2017). To further validate the protective effect of SLBZS on the colonic mucosa, we examined the expression levels of the relevant proteins ZO-1 and occludin. The experimental results showed that the levels of ZO-1 and occludin increased in response to SLBZS treatment, and both were significantly decreased by DSS treatment. These results indicate that SLBZS maintains the intestinal barrier integrity and alleviates the progression of colitis.

In conclusion, SLBZS reduces the production of proinflammatory factors, regulates the MAPK and NF-κB signaling pathways, and inhibits the pyroptosis signaling pathway. In addition, SLBZS also restores colonic tight junction protein expression levels, which maintains the integrity of the colonic mucosal barrier. These results indicate that SLBZS is an effective UC treatment.

## Data Availability Statement

The raw data supporting the conclusions of this manuscript will be made available by the authors, without undue reservation, to any qualified researcher.

## Ethics Statement

The animal study was reviewed and approved by South China Agricultural University.

## Author Contributions

LC, ZL and SG conceived and designed the experiments. LC, ZL, JZ, WC, YL, WL, AG and QQ conducted the experiments. LC and ZL conducted the experiments and wrote the thesis.

## Conflict of Interest

The authors declare that the research was conducted in the absence of any commercial or financial relationships that could be construed as a potential conflict of interest.
